# Ranging in Human Sonar: Effects of Additional Early Reflections and Exploratory Head Movements

**DOI:** 10.1371/journal.pone.0115363

**Published:** 2014-12-31

**Authors:** Ludwig Wallmeier, Lutz Wiegrebe

**Affiliations:** 1 Graduate School of Systemic Neuroscience, Ludwig-Maximilians-Universität München, Planegg-Martinsried, Germany; 2 Division of Neurobiology, Department Biologie II, Ludwig-Maximilians-Universität München, Planegg-Martinsried, Germany; Universität Bielefeld, Germany

## Abstract

Many blind people rely on echoes from self-produced sounds to assess their environment. It has been shown that human subjects can use echolocation for directional localization and orientation in a room, but echo-acoustic distance perception - e.g. to determine one's position in a room - has received little scientific attention, and systematic studies on the influence of additional early reflections and exploratory head movements are lacking. This study investigates echo-acoustic distance discrimination in virtual echo-acoustic space, using the impulse responses of a real corridor. Six blindfolded sighted subjects and a blind echolocation expert had to discriminate between two positions in the virtual corridor, which differed by their distance to the front wall, but not to the lateral walls. To solve this task, participants evaluated echoes that were generated in real time from self-produced vocalizations. Across experimental conditions, we systematically varied the restrictions for head rotations, the subjects' orientation in virtual space and the reference position. Three key results were observed. First, all participants successfully solved the task with discrimination thresholds below 1 m for all reference distances (0.75–4 m). Performance was best for the smallest reference distance of 0.75 m, with thresholds around 20 cm. Second, distance discrimination performance was relatively robust against additional early reflections, compared to other echolocation tasks like directional localization. Third, free head rotations during echolocation can improve distance discrimination performance in complex environmental settings. However, head movements do not necessarily provide a benefit over static echolocation from an optimal single orientation. These results show that accurate distance discrimination through echolocation is possible over a wide range of reference distances and environmental conditions. This is an important functional benefit of human echolocation, which may also play a major role in the calibration of auditory space representations.

## Introduction

Bats and toothed whales are known for using the echoes of self-produced sounds to represent their surrounding environment even in complete darkness, which is referred to as echolocation [1], [2]. There is growing evidence that several other species also use echolocation for navigation and orientation when visual stimuli are not available, including blind humans (for reviews on human echolocation see [3], [4]). It has been shown that blind and blind-folded sighted human subjects can gather spatial information regarding the position [5], [6], [7], [8], size [9], material and shape [10], [11], [12] of a sound reflecting object based on echolocation.

Successful orientation and navigation requires knowledge about the direction and distance of obstacles and landmarks within one's surrounding environment. While there is comprehensive evidence that both blind and sighted humans can use echolocation to detect a sound reflecting surface [12], [13], [14] and to determine its azimuthal direction with high acuity [5], [7], [8], [12], [15], [16], [17], echo-acoustic distance perception has received relatively little scientific attention. The few studies that have investigated the resolution of echo-acoustic distance perception report somewhat conflicting results: Kellogg [18] found that two blind subjects could discriminate differences of 11 and 18 cm in the distance of two consecutively presented sound reflecting surfaces at a reference distance of 61 cm, whereas his blindfolded sighted subjects performed at chance level. In contrast, Schörnich et al. [6] successfully trained five sighted subjects to discriminate differences in distance through echolocation. The reference distances that were tested in this experiment ranged from 1.7 m to 6.8 m. A direct comparison of echo-acoustic distance discrimination for short- compared to long target distances is not available to date. Kellogg [18] reported above chance performance only for blind subjects with close-by reflectors (around 0.6 m distance), whereas Schörnich et al. [6] investigated performance of sighted subjects with reflectors for distances above 1.7 m, only.

There are at least hints that sighted subjects can use echolocation for distance judgment for close-by reflectors as well: in an open-loop walking task, Rosenblum et al. [19] presented sound reflecting surfaces to blindfolded sighted subjects at distances of 0.9 m, 1.8 m, 2.7 m, or 3.7 m. Subjects were able to estimate the distance of the surfaces echo-acoustically with an accuracy of less than 1.2 m. However, the authors point out that it is difficult to compare their results with the results on echolocating distance using other judgment paradigms like in Kellogg's experiment, since the former tested absolute distance perception whereas the latter tested relative distance perception. Indeed, Moore et al. [20] have shown that performances in relative and absolute distance perception tasks are not necessarily coherent. Hence, a comprehensive quantification of the resolution of echo-acoustic distance estimation in humans, which includes performance of blind and sighted subjects both for close-by as well as more distant reflectors, is still missing to date.

This lack is puzzling, since echolocation is the only far sense which allows humans to directly assess distance information, namely by estimating the time delay between sound emission and echo reception. Hence, depth perception may turn out as the primary benefit of echolocation, even more than the perception of object direction, shape or size. Indeed, Kolarik et al. [3] hypothesized that echolocation may help humans to provide a distance reference for internal spatial representations of their acoustic environment. This is a strong motivation for further investigations of echo-acoustic distance perception: Lewald [21] pointed out that the generation and maintenance of these auditory space representations is an “important, but still insufficiently solved, problem in human auditory neuroscience”. Research on echolocating distance may shed new light on this problem.

Anecdotal reports indicate that room reverberation (i.e. echoes from additional reflectors beside the target reflector) and exploratory head movements can improve echo-acoustic distance discrimination performance [18]. Concerning additional echoes, Schörnich et al. [6] found that under certain conditions, detecting changes in the distance to a sound-reflecting surface can be enhanced by the presence of a laterally displaced second reflector. Schenkman and Nilsson [14] also report slightly better echo-acoustic detection performance when the target is placed in an ordinary room as compared to an anechoic chamber. However, a formal quantification of the influence of a subject's position in a room (which determines the reverberation pattern) is not available to date.

Concerning exploratory head movements, enhanced echolocation performance has been shown for the discrimination of 2-D shapes [22] and for directional localization tasks [23], but it has not yet been formally investigated in terms of distance perception. Rosenblum et al. [19] conducted an echo-acoustic distance estimation experiment with human subjects, and found a subtle advantage of linear approaching motion during echolocation as compared to a stationary condition. However, neither distance discrimination acuity nor exploratory head rotations were investigated by Rosenblum et al. [19].

The aim of the current study is to quantitatively describe echo-acoustic distance discrimination performance of human subjects in reverberant rooms. In particular, the objectives are (1) to provide a comprehensive quantification of distance discrimination acuity in blind and sighted subjects both for target reflectors in near space (<1 m) and in far space, (2) to formally quantify the influence of a subject's position in a room as well as (3) the influence of exploratory head rotations on distance discrimination performance. To that end, six blindfolded sighted subjects and one blind echolocation expert performed an echo-acoustic distance discrimination task in virtual echo-acoustic space (VEAS): They actively emitted sounds with their mouths and analysed the echoes that were generated in real time from their own vocalizations. The method of VEAS presentation guaranteed that subjects could not use any other cues beside the intended acoustic ones, and allowed for detailed documentation of head movements.

## Material and Methods

### Subjects

Six sighted subjects participated in the study (24.8±2.9 years of age (mean ± SD), 1 female). All subjects showed hearing thresholds of less than 10 dB HL at both ears for all tested frequencies (250 to 8000 Hz in octave steps). To provide a proof-of-concept for our VEAS presentation and to compare our sighted subjects' performance with that of blind echolocation experts, we also included a blind professional echolocation teacher to our study. The echolocation expert has been blind since infancy, taught himself to echolocate during childhood, and since then has been using echolocation on a daily basis.

The current psychophysical experiments with human subjects have been ethically approved by the Ethikkommission der Medizinischen Fakultät der LMU München (project Nr. 359-07). Subjects signed a written consent form that had been approved by the ethics committee.

### Stimuli and apparatus

**Figure 1:**
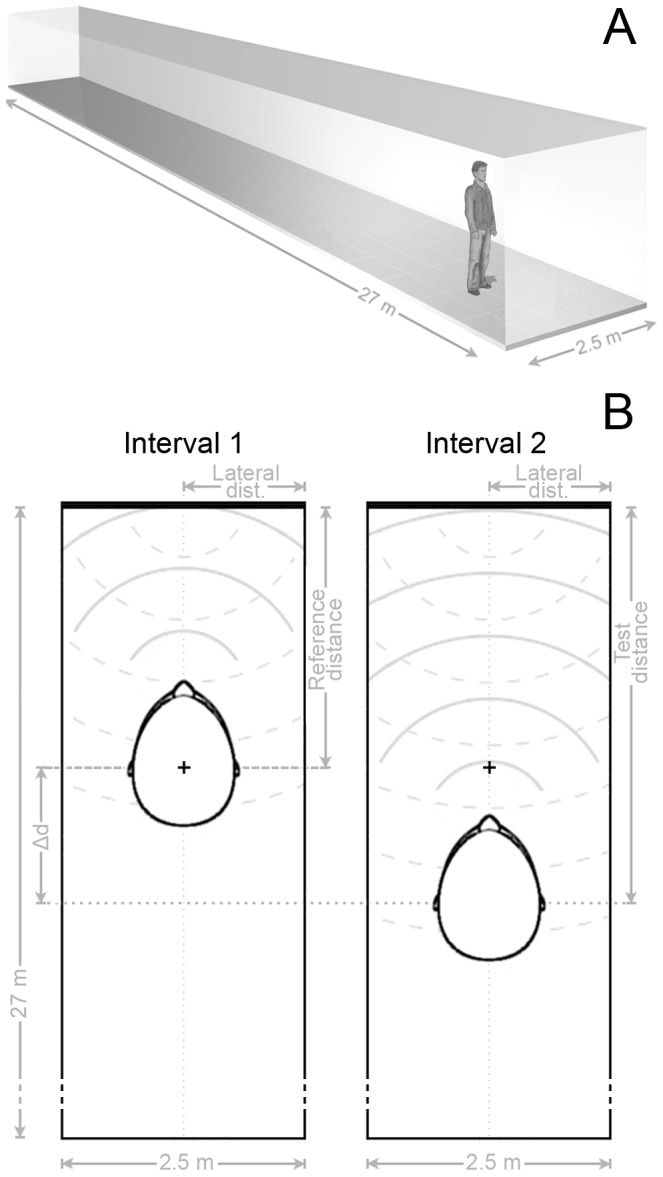
Illustration of the virtual echo-acoustic space (VEAS) and the psychophysical task. (**A**) Illustration of the virtual corridor. All experiments were conducted under headphones and transferred into a virtual echo-acoustic environment, which consisted of a corridor with a length of 27 m and a width of 2.5 m. (**B**) Illustration of the psychophysical paradigm (simplified, drawings of the virtual corridor and the subject's head are not to scale). Subjects gathered spatial information about their environment by listening to echoes (dashed segments of a circle, simplified) of their own vocalizations (continuous segments of a circle). In an adaptive 2AIFC paradigm, two positions in VEAS were consecutively presented which differed in their distance to the front wall (thick, horizontal line), but not in their distance to the lateral walls (vertical lines). The shorter of the two distances to the front wall is called reference distance, the longer is called test distance. The shorter of the two distances to the lateral walls is called lateral distance. The reference distance was randomly changed across trials by ±5% to prevent subjects from memorizing the corresponding sound. Apart from that, the reference position (denoted by the + sign) was held constant during each adaptive track.

In our experiments, subjects gathered spatial information about their environment by listening to echoes of their own vocalizations. All experiments were conducted in VEAS using the binaural room impulse responses (BRIRs) of a real corridor with a constant width of 2.5 m at a length of 27 m and a height of 4 m (cf. **Fig. 1A**). The side walls of the corridor were made of concrete, the flooring consisted of PVC.

The corridor's BRIRs were recorded with the help of a custom built mobile recording setup (which is described in S1 File). Specifically, we acquired a representative database of BRIRs for positions along the midline of the corridor and in close proximity of 65 cm to the lateral walls, with a longitudinal resolution of 25 cm. At each position, measurements were conducted for 90 orientations, i.e. with an angular resolution of 4°. Finally, the BRIRs were interpolated (by linear interpolation of the linear magnitude spectra and unwrapped phase spectra) to increase the angular resolution from 4° to 0.2°, and the longitudinal resolution from 25 cm to 1 cm.

During the experiments, subjects were seated in a sound-attenuated anechoic chamber with a size of 2.0 m×2.0 m×2.2 m (*Industrial Acoustics Company GmbH, Niederkrüchten, Germany*). The walls of the chamber were lined with 20 cm acoustic wedges, which decrease the level of echoes by at least 40 dB at frequencies higher than 500 Hz. A specific position and orientation in VEAS was presented in the following way: the sounds which subjects produced with their mouths were picked up with a headset microphone (*Sennheiser HS2-EW, Wedemark, Germany*), convolved in real time with the respective BRIR, and then presented via headphones (*K701, AKG Acoustics GmbH, Vienna, Austria*). These headphones, although having a circumaural ‘open’ design, still attenuate external sounds. For an echolocation experiment, it is crucial that a subject not only perceives the echoes, but the echoes referenced against the outgoing vocalization. To allow for an optimal sound transmission of the outgoing vocalization into the subject's ears, we removed the circumaural cushions and their mounting such that only the headphone driver remained on each side. Above these drivers, acoustic foam blocks were attached to hold the drivers at a fixed distance of about 3 cm from the head.

The BRIRs presented to the subjects had a length of 2.7 s and were all derived from the BRIRs recorded in the real corridor, while compensating for the frequency-response characteristics of the microphone and the modified headphones. The headphones and the microphone were connected to a personal computer (*PC with Windows 7*) with an external soundcard (*MOTU Audio 24I/O, Cambridge, Massachusetts, USA*) via wireless transmitter and receiver systems (*Sennheiser EW 172 G3 and EW 300 IEM G3, Wedemark, Germany*). On the PC, a real-time convolution kernel (*Soundmexpro, Oldenburg, Germany*) was running under Matlab. The overall input-output delay of the setup was 3.3 ms.

The authentic reproduction of the corridor's acoustics was verified by measuring the BRIRs of the VEAS using the same recording setup and procedure as for the original BRIR acquisition. Additionally, a blind echolocation expert validated the VEAS presentation perceptually (in a formal psychophysical experiment and in an informal comparison of the VEAS and the real corridor).

### General procedure and psychophysical paradigm

In an adaptive two-alternative, two-interval, forced-choice (2AIFC) paradigm with audio feedback, two positions in VEAS were consecutively presented which differed in their distance to the front wall, but not in their distance to the lateral walls (cf. **Fig. 1B**). The order in which the two distances were presented was randomly changed from trial to trial. Subjects were trained to find the interval that contained the shorter of the two distances. In order to keep terminology consistent with previous studies on echo-acoustic distance discrimination, we refer to the shorter distance as 'reference distance' and to the longer distance as 'test distance' like in Schörnich et al. [6].

Each experimental trial started with a 50 ms, 1 kHz tone pip to indicate the beginning of a 5 s exploration interval. During the exploration interval, one of the two positions in VEAS was presented. The ending of the exploration interval was signalled by a 2 kHz tone pip. After a 500 ms pause, the other position was presented in the same way. Subsequently, the subjects had to respond whether the first or the second exploration interval had contained the position that was closer to the front wall. Subjects were given audio feedback in terms of a 250 ms frequency chirp, which was upward modulated when the subjects' response had been correct and downward modulated when the subjects' response had been incorrect.

During an experimental run, the reference distance was randomly changed across trials by ±5% to prevent subjects from memorizing the sound of the presented positions. Apart from that, the reference distance was held constant. The test distance was set to 150% of the reference distance at the beginning of the experimental run. During the adaptive track, the difference Δd between the two distances was changed following a three-down-one-up procedure: it was decreased after three correct responses and increased after one incorrect response, which yields threshold estimates at the 79.4% correct level [24]. Until the third reversal of the adaptive track, Δd was increased or decreased geometrically by a factor of two (i.e., it was halved or doubled), for reversals four and five it was increased or decreased geometrically by a factor of 1.2, and by a factor of 1.1 from the sixth reversal on.

The experimental run was stopped at the eleventh reversal and the just noticeable difference (JND) was calculated as the geometric mean of Δd in centimetres at the last six reversals of the run. All subjects were trained until their performance in terms of JNDs stabilized over runs. The criterion for stable performance was fulfilled when the standard deviation across the last three runs was less than 25% of the mean across these runs. Data acquisition was randomly interleaved across experiments to balance residual training effects.

### Experiment 1: Head-fixed echolocation

**Figure 2:**
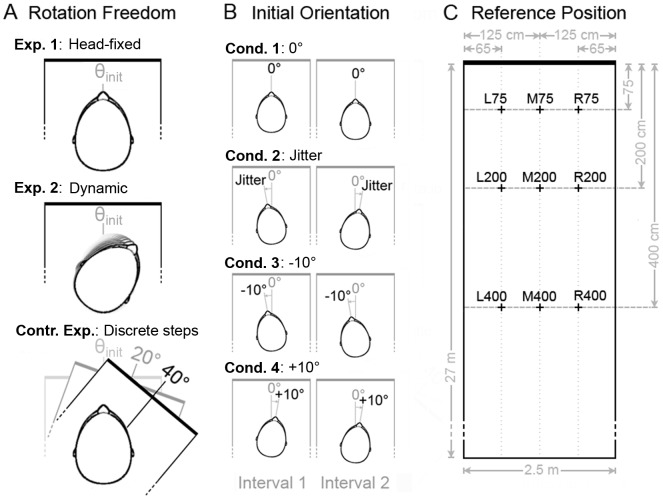
Overview sketch of all experimental conditions tested in this study. (**A**) Three experiments were conducted: In Experiment 1, the subjects' orientation in VEAS was fixed, i.e. the initial orientation (θ_init_) was maintained for the whole course of an exploration interval. In Experiment 2, subjects again started out from θ_init_ but then were free to rotate their heads relative to the VEAS (which was fixed in world coordinates). In the control experiment, subjects' heads and bodies were fixed in world coordinates, but they could rotate the VEAS in discrete 20° steps by pressing a button on a joystick. (**B**) Each experiment was conducted in four different conditions: In condition 1, θ_init_ was always parallel to the VEAS' longitudinal axis (which is illustrated as 0° orientation) in both exploration intervals. In condition 2, θ_init_ was jittered by about ±10° across intervals. In conditions 3 and 4, θ_init_ was always −10° and +10°, respectively. (**C**) Each experiment and condition was conducted at three reference positions along the midline of the corridor (reference positions M75, M200 and M400), at three reference positions near the left lateral wall (L75, L200, L400) and at three reference positions near the right lateral wall (R75, R200, R400).

In Experiment 1, the subjects' orientation in VEAS was fixed, i.e. the initial orientation θ_init_ at the beginning of an exploration interval was maintained during the whole course of the 5 s interval (cf. **Fig. 2A**). In the following, this will be referred to as ‘head-fixed echolocation’.

In order to investigate the influence of the subjects' orientation in VEAS on echo-acoustic distance discrimination performance, there were four different conditions concerning θ_init_ (cf. **Fig. 2B**): In condition 1, θ_init_ was always parallel to the VEAS' longitudinal axis (which is illustrated as 0° orientation in **Fig. 2B**) for both exploration intervals. In condition 2, θ_init_ was randomly jittered to the left and right across intervals, with a maximal absolute deviation of 15° and a mean absolute deviation of 10°. In conditions 3 and 4, θ_init_ was always −10° and +10°, respectively.

In order to investigate the influence of a subject's position in VEAS, there were nine different reference positions (cf. **Fig. 2C**). Specifically, each experimental condition was conducted at three reference positions along the midline of the corridor (reference positions M75, M200 and M400), at three reference positions near the left lateral wall (L75, L200, L400) and at three reference positions near the right lateral wall (R75, R200, R400).

**Figure 3:**
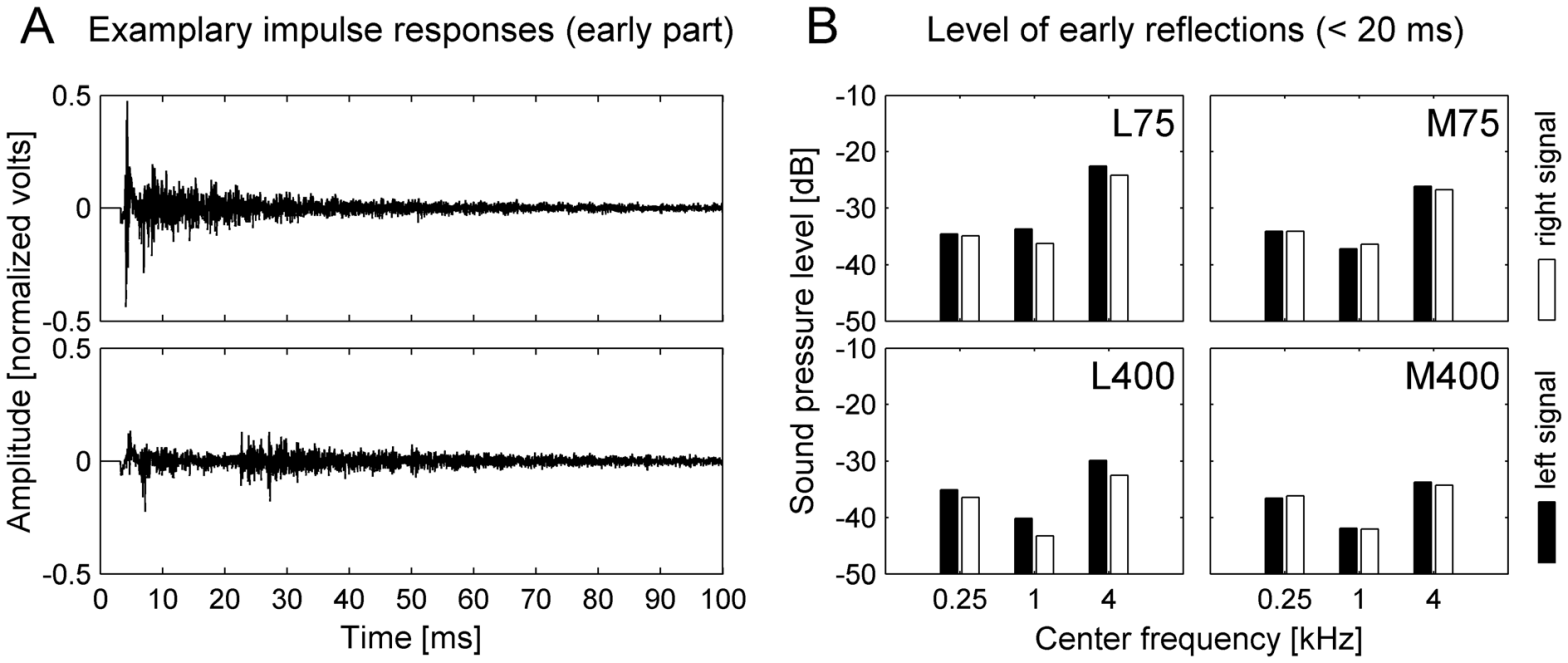
Illustration of the corridor's IRs and how they vary with position. (**A**) Illustration of two exemplary IRs recorded at positions along the corridor's midline (with 0° orientation). The first 3.3 ms of the IRs (which contain e.g. the direct sound from the mouth to the ears) were cut away to account for the input-output delay of the setup. The vertical axis represents the amplitude of the IR in linear units, which was normalized such that the direct sound would have an amplitude of 1 V. Due to the symmetry of the corridor, the left signal and the right signal of the binaural room IR are visually not distinguishable at the resolution and size of the current figure. The figure demonstrates that the pattern of early reflections varies with the distance to the front wall of the corridor: For position M75, there is a peak at 4.4 ms, which represents the echo from the front wall at a distance of 75 cm. For position M400, there is a peak at 23.3 ms, which represents the echo from the front wall at a distance of 4 m. (**B**) Level of the early reflections for three two-octave frequency bands (with center frequencies of 0.25, 1 and 4 kHz). The analysis was conducted for the IRs from four different positions (M75, M400, L75, L400, all at 0° orientation). Only the early part of the IRs within the time interval of 3.3–20 ms was included. The vertical axis represents the effective sound pressure level of the reflections in dB relative to the direct sound (which was set to 0 dB). The figure demonstrates that the proximity to a lateral wall influences the pattern of early reflections: The level of the left ear signal is higher near the left lateral wall than in the middle of the corridor, especially in the high frequency range.

Shinn-Cunningham et al. [25] measured and analysed BRIRs at different positions and orientations in a room. They demonstrated that the position and orientation in a room influences how reverberant energy affects spatial acoustic cues. Especially the early reflections from a nearby lateral wall at about half a meter's distance induced systematic distortions in spatial cues. Analysing the BRIRs used in the current experiments revealed that the distance to the front wall and the distance to the lateral walls of the corridor influence the temporal pattern and the level of early reflections (cf. **Fig. 3**). **Figure 3B** demonstrates that near a lateral wall, the level of early reflections is increased for the ear that faces the lateral wall. These findings and the findings of Shinn-Cunningham et al. show that in our experiments, the different reference positions are appropriate for investigating the influence of additional early reflections from a lateral wall on echo-acoustic distance discrimination performance.

### Experiment 2: Dynamic echolocation

Experiment 2 was designed to investigate the influence of exploratory head-motion on echo-acoustic distance discrimination performance. The same 2AIFC adaptive paradigm as in the head-fixed Experiment 1 was used. However here, subjects were allowed to engage in exploratory head movements during the 5 s exploration intervals (cf. **Fig. 2A**), which will be referred to as ‘dynamic echolocation’. To that end, the orientation of the subject's head was assessed via a tracking system (*Intersense motion tracker, Billerica, Massachusetts, USA*) ten times a second. Depending on the orientation of the subject's head, the respective BRIR was used for VEAS presentation in order to guarantee consistent orientations both in real and in virtual space. BRIRs were updated with the same frequency of 10 Hz. Whenever subjects had rotated their head by at least 1°, the old time-invariant convolution was abruptly stopped and a new convolution process was started with the respective BRIR that corresponds to the new orientation. The subjects' orientations in VEAS were written to a log file and saved to hard disk at the end of each exploration interval.

Like in the stationary Experiment 1, there were the same four conditions concerning the initial orientation θ_init_ (cf. **Fig. 2B**) and the same nine reference positions (cf. **Fig. 2C**). The orientation of the virtual corridor in world coordinates was recalibrated at the beginning of each exploration interval, namely by tracking the subjects' head orientation in real space and defining this to be the initial orientation in VEAS (either 0°, jittered, −10° or +10°).

### Control experiment: Orientation changes in discrete steps

Performance differences between a dynamic condition and a fixed condition may not necessarily result from the dynamic acoustic dimensions available to a moving echolocator, but may be due to the fact that moving subjects are allowed to echolocate from more than one static orientation (Ashmead, 1995; Rosenblum et al., 2000). To address this problem, we conducted a control experiment to Experiment 2. The control experiment was identical to Experiment 2, including the possibility to echolocate with more than one static orientation. However here, orientation changes in virtual space were not mediated by continuous head rotations in real space. Instead, the subjects' head and body was fixed in real space, but they could rotate the VEAS around themselves in discrete steps of 20° by deflecting a joystick either to the left or to the right (cf. **Fig. 2A**). Between two steps, subjects always had to release the joystick to its normal position. This control experiment allows for exploring the spatial layout from different orientations but lacks proprioceptive or vestibular input encoding the change of orientation. In the following, this will be referred to as echolocation with orientation changes in ‘discrete steps’.

The step size of 20° was chosen because a pilot experiment had revealed that the six sighted subjects performed better when the VEAS rotations were conducted in discrete steps of 20°, as compared to discrete steps of 10° or continuous VEAS rotations. With the finer sampling intervals, subjects tended to lose track of their orientation and therefore performed worse.

Again, there were the same four conditions concerning the initial orientation θ_init_ (cf. **Fig. 2B**) and the same nine reference positions (cf. **Fig. 2C**).

### Statistical analyses

The non-parametric Wilcoxon rank-sum test was used to test whether two datasets are independent samples from identical continuous distributions with equal medians. Results are presented in terms of the p-value and the rank-sum statistic W_M,N_, where M and N denote the number of elements in the two datasets. The non-parametric Kruskal-Wallis analysis of variance (ANOVA) was used to compare more than two datasets with respect to a single factor. Results are presented in terms of p-value and test statistic K. Two-way and three-way ANOVAs were used to compare more than two datasets with respect to multiple factors. Results are presented in terms of p-value and test statistic F_D,E_, where D denotes the degrees of freedom of the respective factor and E denotes the degrees of freedom of the error.

Before each analysis, Levene's test was used to assess whether the datasets have equal variances. The assumption of equal variances was rejected when the resulting p-value was less than 0.05. In this case, data were logarithmically transformed for the Wilcoxon rank-sum test and square-root transformed for the ANOVA to provide homoscedasticity (cf. [26]). Due to our stability criterion (standard deviation of less than 25% of the mean), one would expect higher variance for conditions with higher mean JNDs. The data transformations account for this effect. The statistical analysis was then performed both on the original data and on the transformed data. An effect was reported as being significant, if and only if it was significant both for the original data and for the transformed data. This guarantees that the results of all analyses are robust against departures from homoscedasticity, and that they are not influenced by any data transformations.

A one-tailed t-test analysis was used to compare the performance of the blind echolocation expert with the performances of the six sighted subjects (cf. [27]). In Table 1, we report point and interval estimates of the percentage of the control population obtaining a lower JND than the case as described in [28] and for the effect size (z_CC_) for the differences between case and controls as described in [29]. The information presented in Table 1 fully meets the reporting standards set out by Crawford et al. [29].

**Table 1:** Comparison of the blind expert's performance with that of sighted controls.

		*Control sample (n = 6)*		*Case*	*Significance test*		*Estimated percentage of the control population obtaining better JND than the case*		*Estimated effect size (z_CC_)*	
Exp.	Pos.	Mean	SD	JND	t	p	Point	(95% CI)	Point	(95% CI)
1.1	W75	15.3	2.8	9.4	−1.95	0.054	5.43	(0.017 to 27.82)	−2.107	(−3.581 to −0.588)
	W200	38.7	4.1	24.5	−3.21	0.012	1.19	(0.00 to 10.86)	−3.463	(−5.678 to −1.234)
	W400	80.9	6.9	43.0	−5.09	0.0019	0.19	(0.00 to 1.68)	–5.493	(–8.882 to –2.124)
	M75	20.2	2.7	9.3	–3.74	0.0067	0.67	(0.00 to 6.79)	–4.037	(–6.579 to–1.491)
	M200	36.5	4.5	19.0	–3.60	0.0078	0.78	(0.00 to 7.70)	–3.889	(–6.346 to –1.425)
	M400	68.9	4.7	38.0	–6.09	0.00087	0.087	(0.00 to 0.49)	–6.574	(–10.602 to –2.583)
1.2	W75	29.6	5.5	17.1	–2.10	0.045	4.46	(0.006 to 25.09)	–2.273	(–3.833 to –0.672)
	W200	58.3	7.1	35.1	–3.03	0.015	1.46	(0.00 to 12.61)	–3.268	(–5.372 to –1.145)
	W400	102.5	7.9	57.5	–5.27	0.0016	0.16	(0.00 to 1.35)	–5.696	(–9.205 to –2.211)
	M75	28.2	5.2	14.4	–2.46	0.029	2.87	(0.00 to 19.56)	–2.654	(–4.418 to –0.857)
	M200	47.5	4.6	27.8	–3.97	0.0054	0.53	(0.00 to 5.48)	–4.283	(–6.967 to –1.600)
	M400	78.9	6.7	46.8	–4.44	0.0034	0.34	(0.00 to 3.42)	–4.791	(–7.770 to –1.822)
2.1	W75	15.2	2.9	9.8	–1.72	0.073	7.27	(0.066 to 32.21)	–1.862	(–3.212 to –0.462)
	W200	39.2	6.3	21.7	–2.57	0.025	2.50	(0.00 to 17.97)	–2.778	(–4.610 to –0.917)
	W400	69.4	6.8	42.2	–3.70	0.0070	0.70	(0.00 to 7.01)	–4.000	(–6.521 to –1.475)
	M75	18.7	3.6	9.5	–2.37	0.032	3.21	(0.00 to 20.89)	–2.556	(–4.266 to –0.810)
	M200	38.5	5.0	19.3	–3.56	0.0082	0.81	(0.00 to 8.02)	–3.840	(–6.269 to –1.404)
	M400	71.9	8.7	39.1	–3.49	0.0087	0.87	(0.00 to 8.50)	–3.770	(–6.159 to –1.372)
2.2	W75	20.5	4.5	10.6	–2.04	0.049	4.86	(0.010 to 26.27)	–2.200	(–3.722 to –0.635)
	W200	45.3	5.5	26.8	–3.11	0.013	1.32	(0.00 to 11.73)	–3.364	(–5.522 to –1.189)
	W400	76.8	8.0	45.4	–3.63	0.0075	0.75	(0.00 to 7.47)	–3.925	(–6.403 to –1.442)
	M75	21.9	4.5	9.9	–2.47	0.028	2.83	(0.00 to 19.39)	–2.667	(–4.438 to –0.864)
	M200	42.1	4.9	22.9	–3.63	0.0076	0.75	(0.00 to 7.51)	–3.918	(–6.392 to –1.439)
	M400	72.1	8.3	39.8	–3.60	0.0078	0.77	(0.00 to 7.68)	–3.892	(–6.350 to –1.427)

The blind echolocation expert performed better than the sighted subjects in all experimental conditions, especially for large reference distances. The table shows results of a one-tailed t-test analysis (cf. [27]) comparing the performance in terms of JND of the blind echolocation expert with the performance of the six sighted subjects. It includes reporting point and interval estimates of the percentage of the control population obtaining a better JND than the case as described in [28] and for the effect size (z_CC_) for the differences between case and controls as described in [29].

## Results

**Figure 4:**
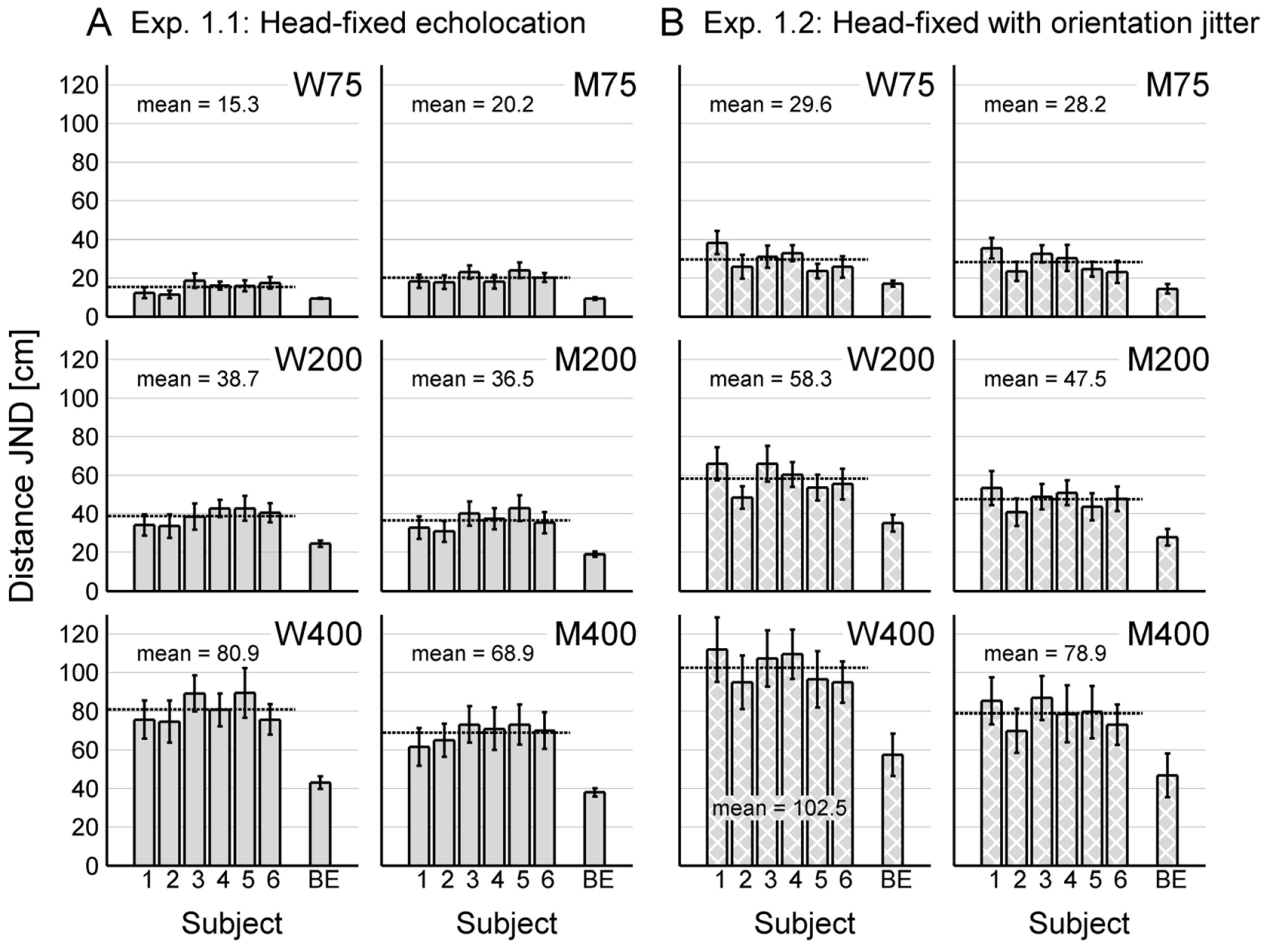
Results of Experiment 1. Performance is shown in terms of individual JNDs (averaged across the last three experimental runs for each subject, error bars represent standard deviations across these runs) for the six sighted subjects (subject 1–6) and the blind echolocation expert (BE). The mean performance across all sighted subjects is displayed as a dashed line and in written form. Panels in the same row represent the same reference distance, panels in the same column represent the same lateral distance (M75, M200, M400: results from positions along the corridors midline; W75, W200, W400: averaged results from positions near the left and near the right lateral wall). (**A**) Without orientation jitter, the sighted subjects performed best at reference position W75 and worst at reference position W400. This shows that a nearby lateral wall can act as a helpful reference when the distance to the target reflector and to the lateral reflector are approximately in the same range, whereas it impedes performance when the target distance exceeds the distance to the lateral wall considerably. (**B**) Introducing an orientation jitter impaired performance significantly in close proximity of a lateral wall (W75, W200, W400), especially for large reference distances. The blind echolocation expert performed better than the sighted subjects in all conditions, especially for large reference distances.

**Figure 5:**
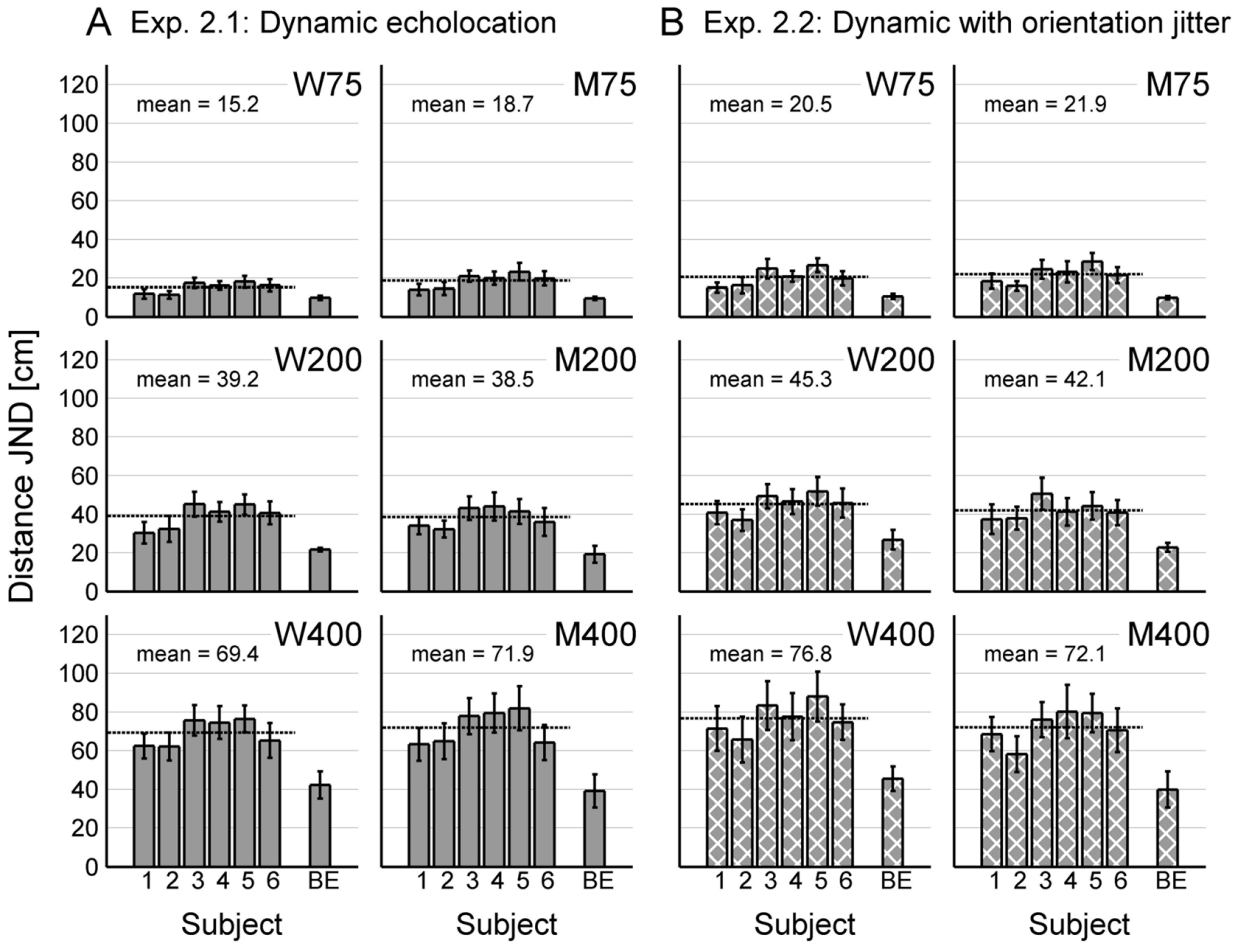
Results of Experiment 2. Performance is shown in terms of individual JNDs (averaged across the last three experimental runs for each subject, error bars represent standard deviations across these runs) for the six sighted subjects (subject 1–6) and the blind echolocation expert (BE). The mean performance across all sighted subjects is displayed as a dashed line and in written form. Panels in the same row represent the same reference distance, panels in the same column represent the same lateral distance (M75, M200, M400: results from positions along the corridors midline; W75, W200, W400: averaged results from positions near the left and near the right lateral wall). (**A**) Without orientation jitter, subjects did not profit from the additional head rotations: Only at reference position W400, performance was significantly better in Exp. 2.1 than in Exp. 1.1. However, the control experiment showed that here subjects did not exploit the additional dynamic dimension, but rather turned their head away from the close-by lateral wall and then echolocated at this static orientation. (**B**) In contrast to Exp. 1 and the control experiment, introducing an orientation jitter did not impair performance for any reference position in Exp. 2. This shows that free head rotations helped subjects to overcome the negative effect of orientation jitter. Again, the blind echolocation expert performed better than the sighted subjects in all conditions, especially for large reference distances.

**Figure 6:**
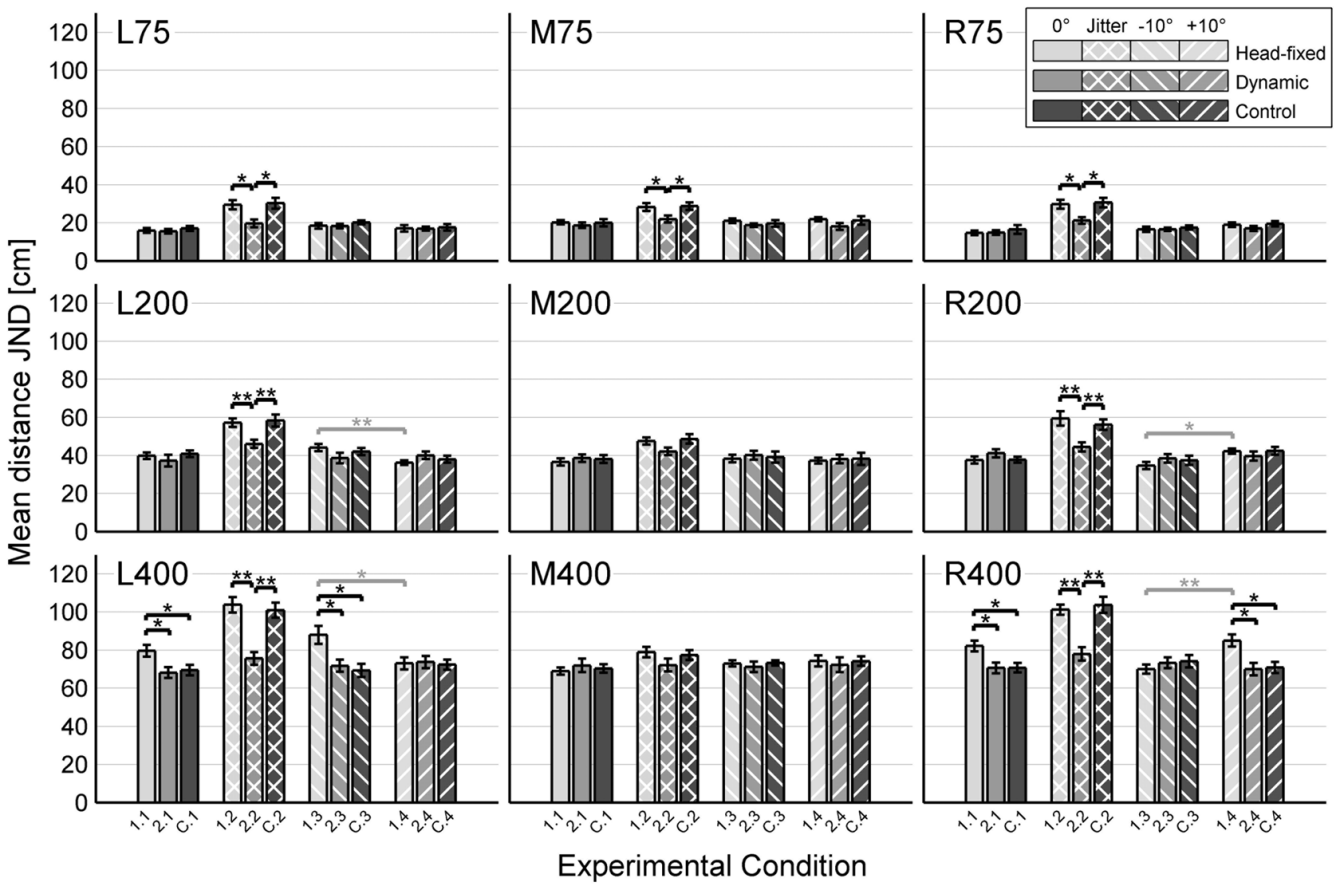
Results of all distance discrimination experiments. Performance is shown in terms of mean JNDs (averaged across six sighted subjects, error bars represent standard errors of the means), plotted against experimental conditions. Each panel represents one reference position (cf. Figs. 1, 2C). Experiments are encoded by different grey scales (light grey, grey and dark grey for Exp. 1, Exp. 2 and the control experiment, respectively). Conditions are encoded by different stripe patterns (no stripes for 0° initial orientation; stripes to the left or right for initial orientation of 10° to the left or right, respectively; stripes both to the left and right for jittered initial orientation). The same grey-scale- and stripe-pattern codes are used throughout this paper. Additionally, the horizontal axes are labelled with abbreviations for the respective experimental conditions (cf. Fig. 2A, B). These labels are redundant to the grey-scale- and stripe-pattern codes, however they might be convenient for the reader since the abbreviations are used in the text to refer to specific experimental conditions. The stars highlight significant differences between pairs of experimental conditions (Wilcoxon-rank-sum test, * for p<0.05, ** for p<0.01). There were no significant differences between the non-jittered conditions (0°, −10°, +10°) and there were no significant differences between the left side (L75, L200, L400) and the right side (R75, R200, R400), except from experiments 1.3 and 1.4 at reference distances of 2 and 4 m. These exceptions are marked with grey significance bars.

All six sighted subjects were successfully trained to perform the echo-acoustic distance discrimination experiments. Results from Experiments 1 and 2 in terms of individual JNDs for all subjects are shown in **Figs. 4** and **5**, respectively. **Fig. 6** gives a comprehensive overview of the average JNDs across sighted subjects for all experimental conditions. This allows for a direct comparison of the subjects' echolocation performances under the head-fixed condition, under the dynamic condition, and under the control condition.

**Fig. 6** shows that there were generally no significant differences between reference positions near the left lateral wall (L75, L200, L400) and the respective positions near the right lateral wall (R75, R200, R400). This was expected because of the symmetry of the virtual corridor. To avoid redundancy, results for reference positions near the left lateral wall and near the right lateral wall were combined and averaged for each subject. In **Figs. 4** and **5**, we present data for positions along the corridor's midline (M75, M200, M400) and the combined data for positions near a lateral wall (W75, W200, W400).

In the current experiments, subjects were allowed to choose any kind of echolocation call, as long as they produced it with the mouth. After a few training sessions, all subjects ended up emitting short tongue clicks, and continued to do so during the whole data acquisition. The clicks typically had a duration between 2 and 8 ms. The sound pressure levels ranged from about 75 to 95 dB SPL as measured at the headset microphone. The clicks had a relatively high peak frequency of around 2 to 8 kHz. The cut-off frequencies at −15 dB below and above the peak frequency (high-pass and low-pass frequencies) were around 1 to 5 kHz and around 4 to 12 kHz, respectively. Further details on the sound analysis are available in S1 File.

### Experiment 1: Head-fixed echolocation

Experiment 1 aimed to formally quantify the influence of reference position and orientation on echo-acoustic distance discrimination in humans. The panels on the right side of **Fig. 4A** show the subjects' performance for positions along the virtual corridor's midline (M75, M200 and M400), i.e. when both lateral walls were symmetrically arranged at the maximum distance of 125 cm. Here, subjects could detect changes in distance to the front wall of around 20 cm for a reference distance of 75 cm. This provides evidence that sighted people are able to use echolocation for distance discrimination not only in far space (as demonstrated by [6]), but also in near space.

However, both the JNDs and inter-subject variability increased with increasing reference distance: Subjects could discriminate differences of around 30–45 cm at a reference distance of 2 m, and of around 60–80 cm at a reference distance of 4 m. A Kruskal-Wallis nonparametric ANOVA showed a significant effect of reference distance (K = 15.16, p<0.001). This confirms previous studies reporting echolocation performance to deteriorate with increasing reflector distance [18], [30], [31].

The effect of reference distance was even more pronounced for positions in close proximity of a lateral wall: The panels on the left side of **Fig. 4A** show that a nearby lateral wall induced a significant improvement in performance for the smallest reference distance of 75 cm (Wilcoxon-rank-sum test, W_6,6_ = 24, p = 0.015). Here, the average JND was as low as 16 cm. On the other hand, it significantly impaired performance for the largest reference distance of 4 m (W_6,6_ = 57, p = 0.0022), resulting in an average JND of about 80 cm. The negative effect of a nearby lateral wall at 4 m reference distance occurred for initial orientations of 0° and -10° at L400 and for initial orientations of 0° and +10° at R400 (cf. **Fig. 6**). This shows that a nearby lateral wall acts as interfering masker when the target distance considerably exceeds the lateral distance, except for orientations away from the nearby lateral wall. For the medium reference distance of 2 m, there was no significant difference between performances in the middle of the corridor and near a lateral wall (W_6,6_ = 44, p = 0.48).

For Experiment 1.1, a two-way ANOVA on all reference positions shown in **Fig. 4A** revealed a significant main effect of reference distance (F_2,30_ = 498.18, p<0.001) and a significant interaction effect of reference distance and lateral wall distance (F_2,30_ = 10.63, p<0.001), but no significant main effect of lateral wall distance (F_1,30_ = 4.12, p = 0.051). The same ANOVA was performed on the square-root transformed data to provide homoscedasticity. With the transformed data, the effect of reference distance (F_2,30_ = 511.90, p<0.001) and the interaction effect of reference distance and lateral distance (F_2,30_ = 10.71, p<0.001) were still significant, and the effect of lateral distance (F_1,30_ = 0.63, p<0.43) was still insignificant. This shows that the results of the ANOVA are robust against departures from homoscedasticity.

Introducing an orientation jitter in Experiment 1.2 impaired distance discrimination performance for all subjects (cf. **Fig. 4B**). Wilcoxon-rank-sum tests revealed significantly worse JNDs in Experiment 1.2 than in Experiment 1.1 for all reference positions (p<0.05), except for reference position M400 (W_6,6_ = 27, p = 0.065). The negative effect of orientation jitter was especially pronounced in close proximity of a lateral wall: A three-way ANOVA with the factors jitter (two levels), reference distance (3 levels) and lateral distance (two levels) showed a significant main effect of orientation jitter (F_1,62_ = 122.07, p<0.001) and a significant interaction effect of orientation jitter and lateral distance (F_1,62_ = 12.06, p<0.001). The same ANOVA was performed on the square-root transformed data to provide homoscedasticity. With the transformed data, the effect of orientation jitter (F_1,62_ = 135.02, p<0.001) and the interaction effect of orientation jitter and lateral distance (F_1,62_ = 11.39, p = 0.0013) were still significant. This shows that the results of the ANOVA are robust against departures from homoscedasticity. As a consequence, performance in Experiment 1.2 was worse near the lateral wall than along the corridor's midline: In contrast to Experiment 1.1, there was a highly significant main effect of lateral wall distance in Experiment 1.2 (F_1,30_ = 32.38, p<0.001).

The blind echolocation expert successfully performed the echo-acoustic distance discrimination experiment without any specific training (cf. **Fig. 4**), which provides a proof-of-concept for our VEAS presentation. His JND was best at reference position M75. Here, he was able to detect changes in distance to the front wall of about 10 cm. Performance decreased with increasing reference distance and in proximity of the lateral wall. In contrast to the sighted subjects, the blind expert did not profit from a nearby lateral wall at any reference distance. However, the blind expert still performed better than the sighted subjects – who had received extensive amounts of training in each specific task before data acquisition - in all conditions of Experiment 1. Especially at large reference distances, his performance was significantly better than that of the sighted subjects (cf. **Table 1**). This indicates that blind people are able to use echolocation for precise distance discrimination not only in near space (as demonstrated by [18]), but also in far space.

### Experiment 2: Dynamic echolocation

Experiment 2 aimed to formally quantify the influence of exploratory head rotations on echo-acoustic distance discrimination in humans. Results are shown in **Fig. 5** in terms of individual performances, as well as in **Fig. 6** in terms of mean performances across sighted subjects together with the respective results from Experiment 1 and from the control experiment, which allows for direct comparison of results across experiments.

Without orientation jitter, there was no significant effect of head rotation on distance discrimination performance: Wilcoxon-rank-sum tests showed that results in Experiment 2.1 are not significantly different from results in Experiment 1.1 for any reference position, except for reference position W400. Here, performance was significantly better when head rotations were allowed (W_6,6_ = 53, p = 0.026). Note that this was the only reference distance for which we had observed a significantly negative effect of the nearby lateral wall in Experiment 1.1. This negative effect was absent in Experiment 2.1 (W_6,6_ = 33, p = 0.39), which indicates that head rotations helped subjects to compensate for the masking effect of the echo from the lateral wall imposed on the target echo from the front wall. Analysing the subjects' head orientations over time – which were tracked and saved to a log file – showed that subjects had turned their head away from the nearby lateral wall in this condition, rather than engaging in exploratory head rotations from side to side. Importantly, the blind echolocation expert pursued this strategy as well, which indicates that turning the head was indeed more helpful than exploratory head rotations in this task. Moreover, there was no significant performance difference between Experiment 2.1 and the control experiment C.1 at reference position W400 (W_6,6_ = 36, p = 0.70). In the control experiment, subjects had switched their orientation away from the nearby lateral wall at maximum reference distance. This behaviour takes the same effect as the head turns in Experiment 2.1, namely the possibility to echolocate from a single orientation that offers minimum interference of the lateral reflector. The current results indicate that in a distance discrimination task, exploratory head movements do not necessarily provide a benefit over static echolocation from an optimal single orientation. Results from conditions 3 and 4 with initial orientations towards and away from the nearby lateral wall, respectively, provide additional evidence for this conclusion (cf. **Fig. 6**).

With orientation jitter, however, there was a significantly positive effect of head rotation on distance discrimination performance for most reference positions (cf. **Fig. 6**): Results in the dynamic Experiment 2.2 are significantly better than results in the head-fixed Experiment 1.2 and in the multi-stationary control experiment C.2 for all reference positions near a lateral wall and M75 (Wilcoxon-rank-sum tests, p<0.05). The latter comparison shows that in Experiment 2.2, judgements were indeed enhanced because of the dynamic acoustic changes due to motion, and not because subjects were allowed to echolocate from more than one static orientation. The improvement due to head rotation was especially pronounced for reference positions W200 (W_6,6_ = 55, p = 0.0087) as well as W400 (W_6,6_ = 57, p = 0.0022), i.e. under those conditions for which the impairment due to orientation jitter was maximal in Experiment 1.2. In contrast to Experiment 1, there was no significant difference between Experiment 2.1 without orientation jitter and Experiment 2.2 with orientation jitter for any reference position (Wilcoxon-rank-sum tests, p>0.05 for each reference positions). Hence, the results from the current Experiment 2 indicate that free head rotations during echolocation helped subjects to compensate for the negative effect of orientation jitter.

Again, the blind echolocation expert performed better than the sighted subjects in all conditions of Experiment 2, especially at large reference distances (cf. **Table 1**).

## Discussion

The current psychophysical experiments show that sighted subjects can be successfully trained to gain spatial information about their position in a virtual room through active echolocation, and detect changes in the distance to the room's walls. Distance discrimination JNDs of the sighted subjects were around 20 cm for a reference distance of 75 cm and the maximum lateral wall distance. Performance declined with increasing reference distance. This effect was reinforced in close proximity of a lateral wall, i.e. a close-by lateral wall induced a relative improvement in performance for the minimum reference distance and a relative decline in performance for the maximum reference distance. The blind echolocation expert did not profit from the close-by lateral wall at any position, however he still outperformed the sighted subjects in all conditions, especially for large reference distances. Introducing an orientation jitter impaired distance discrimination performance for all subjects, especially in close proximity of a lateral wall. The negative effect of orientation jitter could be compensated for when additional head rotations were allowed.

### Validity of methods and results

All experiments were transferred into virtual echo-acoustic space (VEAS), using the binaural room impulse responses (BRIRs) of a real corridor. This technique allows for strict experimental control of stimulus parameters and detailed documentation of the sensory-motor interactions underlying human echolocation. The validity of our VEAS implementation was verified both physically and psychophysically: First, we measured and compared BRIRs in real and in virtual space. Second, a professional blind echolocation expert evaluated our VEAS presentation perceptually.

Moreover, some conditions of the current study are comparable to previous experiments on echo-acoustic distance discrimination in real and in virtual space, which allows controlling the validity of our methods through checking the consistency of results. Kellogg [18] conducted a distance discrimination experiment that is roughly similar to our condition with head rotations at reference position M75. He observed – but did not control – the use of head rotations during echolocation. The two blind subjects in his experiment could reliably detect changes in target distance of 11 and 18 cm, respectively, at a reference distance of 61 cm. These results are highly consistent with the JNDs of 19 and 10 cm that our sighted subjects and the blind expert obtained in the respective condition of the current study. Furthermore, Schörnich et al. [6] trained five sighted subjects to detect changes in the distance of a reflective surface through echolocation. Most of their subjects could detect changes of 30–40 cm at the smallest tested reference distance of 1.7 m, which is consistent with the JNDs of around 30–45 cm that we observed in the static Experiment 1.1 at reference position M200.

Furthermore, several authors report that echolocation performance decreases with increasing distance of the target reflector [18], [30], [31], which again agrees with our findings. Concerning auditory distance perception with external sound sources, Zahorik et al. [32] provided a summary of the literature which suggests that changes of about 10–20% in sound source distance are just noticeable for absolute distances of approximately 0.5–5 m. This is again consistent with our observation that JNDs in reflector distance increase with increasing absolute distance. Hence, these studies provide evidence for the validity of our methods and of our results.

Our BRIRs were recorded with the constant directivity of the B&K 4128C head-and-torso simulator. In contrast, human echolocators may change the directivity of their vocalisations either deliberately or as a byproduct of e.g. timbre changes. While in our VEAS implementation, timbre changes would be maintained, the VEAS would assume that these timbre changes do not affect the frequency-dependent directivity. Nothing is known about the extent to which human echolocators do recruit changes in vocalisation directivity as such. However our tests with blind human echolocation experts confirm that these humans could do complex orientation and navigation tasks in our VEAS without relying on such changes of directivity. So overall, while our VEAS implementation cannot capture changes vocalisation directivity, it appears that the VEAS is perceptually plausible enough even for blind echolocation experts, to readily use it.

### Possible cues for echo-acoustic distance discrimination

Echo-acoustic distance perception can be based on a variety of acoustic cues and their combinations. Possible cues that have been proposed in the echolocation literature [3] include sound intensity, time delay between direct sound and echo, and comb filtering effects encoding this time delay.

In our echolocation experiments, JNDs in reflector distance increased with increasing absolute distance. This suggests that our subjects have used intensity cues to reflector distance: Under idealised conditions with intensity as the only cue to sound source distance and an inverse-square law relationship between sound intensity and source distance, the JND for sound source distance would be around 10% of the absolute distance [32]. In an echolocation context, the sound always travels twice the distance between the subject and the reflector. Hence, JNDs for echolocating distance based on intensity cues only should be around 20%. Our data are roughly consistent with this value, which indicates that echo intensity was an important cue to reflector distance in our experiments.

The blind echolocation expert and some of the sighted subjects had even lower JNDs than predicted based on intensity cues only. This suggests that they used additional cues such as the time delay between direct sound and echo. This time delay might be perceived as such or in terms of comb filtering effects [3]. Indeed, for some conditions there was a temporal overlap of the direct sound and the echo: Subjects typically produced tongue clicks with a duration of 3–8 ms. The minimum wall distance was 65 cm which corresponds to an echo delay of 3.8 ms. The temporal overlap will cause changes in the timbre or pitch of the stimuli arriving at the subjects' ears. The time delay between direct sound and echo produces a spectral interference pattern with a fundamental frequency that is inversely proportional to the reflector distance [33]. Since humans are highly sensitive to changes in fundamental frequency [34], [35], the comb-filtering effect might serve as a cue for echo-acoustic distance perception.

However, given the relatively high peak frequencies and high-pass frequencies of the subjects' vocalizations, it is unlikely that subjects relied on comb-filtering effects between direct sound and echo as a primary cue to reflector distance. In the current experiments, the interference pattern would have a relatively low fundamental frequency of maximally 263 Hz (the reciprocal of 3.8 ms). In contrast, the echolocation clicks had high peak frequencies of 2–8 kHz and high-pass frequencies of at least 1 kHz. Hence, comb-filtering effects in the low frequency range would not be very salient.

### The influence of room reverberation or additional echoes

Most previous studies on human echolocation have focussed on the perception of a single reflector. However, realistic everyday environments usually contain more than one sound reflecting surface. Hence, additional reverberant energy may interfere with the perception of a target echo. In order to investigate the influence of additional reverberant energy on echo-acoustic distance discrimination, we varied the subjects' distance to the lateral walls of the virtual corridor. Specifically, each experiment was conducted at a lateral wall distance of 125 cm (which corresponds to the midline of the corridor and therefore is maximal) and at a lateral wall distance of 65 cm (which corresponds to an echo delay of 3.8 ms and therefore is close to the minimum delay of our setup). Previous research has shown that lateral walls produce a salient echo only within a range of about 1 m: Ashmead et al. [36] found that the lateral walls of a hallway provide echo-acoustic information which is useful to guide locomotion, but only within less than a meter's distance from the walls. Moreover, Shinn-Cunningham and Ram [37] measured head-related transfer functions (HRTFs) with a KEMAR manikin in a classroom for different positions of the manikin and for different positions of the sound source. The HRTFs were convolved with pseudo-random white noise bursts and presented to human subjects via headphones in order to simulate different positions in a room in the presence of a sound source radiating white noise. Subjects robustly perceived lateral walls at a distance of half a meter and were able to use this information to identify their position in the classroom. These findings indicate that in our experiments, the lateral wall produced a salient echo at the lateral positions but much less so along the corridor's midline. Hence, one might expect important information to be masked by this task-irrelevant acoustic energy and therefore performance to be worse at the lateral positions. Note that the target echo from the front wall arrives later at the subjects' ears than the echo from the close-by lateral wall for all reference distances in the current experiments. Such a stimulus configuration is known to favour the perception of the first arriving wave front at the expense of the later arriving one due to the precedence effect [38], [39].

However, results from the current Experiment 1.1 show that the echo from a nearby lateral wall not necessarily acts as an interfering masker which impedes the perception of the target reflector. At short reference distances, most subjects even performed better in close proximity of the lateral wall (positions L75 and R75) than in the middle of the corridor (position M75). Here, subjects seem to have used the echo from the lateral wall as a temporal reference when assessing the delay of the target echo. Thereby, they might have exploited differences in the binaural and timbre characteristics of the two echoes. The fact that performance at positions L75 and R75 decreased when an orientation jitter was introduced confirms the importance of directional, binaural cues. Moreover, comb filter effects might have served as a distance cue as well. Note that for positions L75 and R75 there was a temporal overlap of the echo from the lateral wall and the echo from the front wall. The overlap of the two echoes will alter the spectral characteristics of the resultant stimuli arriving at the ears, as argued above for overlapping direct sound and echo. Indeed, the temporal separation between the two echoes at positions L75 and R75 is much smaller than between the direct sound and the echoes. Specifically the distance difference between the lateral and front walls is on the order of 10 cm. Thus, the resulting interference pattern will have a fundamental frequency around 1700 Hz which could potentially create salient pitch cues. However, it is well known that the human binaural system suppresses pitches arising from laterally displaced echoes (e.g. [40]). Thus, we consider the use of pitch cues unlikely also under these experimental conditions.

The observed positive effect of a nearby lateral wall is consistent with the results of Schörnich et al. [6], who report that detecting changes in the distance to a sound-reflecting surface can be enhanced by the presence of a laterally displaced second reflector. Note that Schörnich et al. [6] simulated a reflecting surface at a specific position in virtual space by convolving the subjects' vocalizations with the subjects' head-related impulse response corresponding to the respective direction. A multi-reflector arrangement was simulated by summing up the respective impulse responses before the convolution. This is a simplification compared to natural conditions, since the simulated reflections are point shaped without spatial extent and interference between echoes is limited. For the current experiments, the BRIRs of a real environment were recorded, which take into account the spatial extent of all reflective surfaces as well as realistic interference between echoes. Our Experiment 1.1 shows that the results of Schörnich et al. [6] hold true under more realistic conditions, which confirms that their simplifications are justified.

The finding that echo-acoustic distance perception can be enhanced by the presence of a laterally displaced second reflector supports the ‘information-surplus principle’ suggested by Schenkman and Nilsson [14]: this principle states that task-irrelevant reflections - which would be considered interference for other forms of audition - can provide helpful cues for echolocation, rather than impede performance by masking important information. An example for this principle is given by Schenkman and Nilsson: their subjects were better at detecting a target reflector when the target reflector was placed in an ordinary room than when it was placed in an anechoic chamber. In a listening task, one would expect that it is more difficult to detect a target sound source in the presence of additional, competing sound sources than in the absence of additional sound sources. In Schenkman's and Nilsson's echolocation experiment, however, subjects performed better in the presence of additional reflections than in the absence of additional reflections.

The information-surplus principle somewhat differs from what might be expected based on masking and precedence phenomena in the context of the localization of external sound sources. Indeed, Wallmeier et al. [5] have shown that the classically described asymmetry in the perception of directional information in leading and lagging sounds is diminished in an echolocation context. However in their experiment, it was still more difficult to assess directional information of a target echo in the presence of additional echoes as compared to a single-reflector condition, whereas the current Experiment 1.1 shows that the availability of distance information is much less affected or even enhanced in close proximity of a lateral reflector. This indicates that the effect of additional echoes on echolocation performance strongly depends on the specific task: the information-surplus principle seems to hold true for distance estimation, but not for directional localization, i.e. additional reflections can enhance echo-acoustic distance perception, whereas they impede directional echolocation. This can be explained in the following way: For directional localization, interaural level differences are important. Shinn-Cunningham et al. [25] have shown that "reverberation reduces the magnitude of interaural level differences at all frequencies". This indicates that additional reflections may also impede directional localization of a target echo. On the other hand, the direct-to-reverberant ratio serves as a cue to sound source distance (for a review see [41]). This indicates that additional reflections may also act as a helpful reference for echo-acoustic distance perception.

Only for the maximum reference distance, our subjects performed worse when they were near the lateral wall (positions L400 and R400) than in the middle of the corridor (position M400).

Note that for reference positions L400 and R400, the reference distance exceeds the minimum lateral wall distance by a factor of six. Under idealized conditions with point-shaped sound sources in a free field, the inverse distance law would imply a drop in sound level of 15.5 dB for an increase in distance by a factor of six. Indeed, **Fig. 3B** shows that the echo from the nearby lateral wall induces an increase in the level at position L400 relative to position M400, especially for the ear that faces the nearby lateral wall. Although this increase in level is less pronounced than it would be under idealized conditions, it indicates that the observed effect of relative distance to the target reflector and to the lateral reflector most likely results from energetic masking of the target echo. Results from Experiment 2.1 and the control experiment show that this masking effect can be reduced by turning the head away from the close-by lateral wall. Conditions 3 and 4 with initial orientations either towards or away from the close-by lateral wall provide further evidence for this conclusion.

### The influence of exploratory head rotations

The hypothesis that exploratory head rotations could enhance echo-acoustic distance perception in humans goes back to Kellogg [18]: He reported that his blind subjects spontaneously executed periodic yaw movements of their heads during an echo-acoustic distance discrimination task. He observed that the blind subjects, who did engage in this kind of acoustic scanning behaviour, outperformed the sighted subjects, who did not. Kellogg concluded that head “oscillations enhance the accuracy of perceiving the target” reflector in a distance discrimination task. However, Kellogg did not compare echolocation performance with and without head rotations in the same subjects. Moreover, the experiment was conducted under conditions that do not allow controlling the orientation of the subjects' heads relative to the target reflector in full detail.

In contrast to Kellogg's experimental setup, our VEAS technique allows for investigating echolocation performance both with precisely fixed orientation and with orientation jitter. With orientation jitter, performance was significantly better in the dynamic Experiment 2.2 than in the head-fixed Experiment 1.2 and the multi-stationary control experiment C.2. This performance difference might result from the fact that the resolution of the VEAS was higher in Experiment 2.2 than in the other experiments. However, subjects were not able to exploit higher resolutions of the VEAS without self-motion in the pilot experiment. Hence, the current experiments show that head rotations do indeed enhance distance discrimination performance, at least with orientation jitter. Self-motion seems to have helped subjects to combine and benefit from several orientations, whereas in the control experiment they just selected one best orientation.

Without orientation jitter, distance discrimination performance was significantly better than with orientation jitter. Under these conditions, however, head rotations did not provide an additional benefit over stationary echolocation in the distance discrimination task: performances in the head-fixed Experiment 1.1 and the dynamic Experiment 2.1 did not differ significantly, except for one reference position. A control experiment revealed that even for this position, dynamic head rotations did not provide a benefit over static echolocation from an optimal orientation. This indicates that exploratory head rotations do not enhance echo-acoustic distance discrimination directly, but indirectly through helping subjects to compensate for the negative effect of orientation jitter.

Hence, analysing in which way orientation jitter impairs echo-acoustic distance discrimination may also shed light on the influence of exploratory head rotations on echolocation. Orientation jitter produces variations in echo-acoustic cues on top of the variations due to distance changes. Because of the orientation uncertainty, subjects were not able to completely disentangle whether variations originated from distance changes or from orientation changes. Therefore, echo-acoustic distance cues were partially masked and performance deteriorated. The negative effect of orientation jitter on distance discrimination performance was especially pronounced in close proximity to a lateral wall. This is no surprise, because even small changes in orientation can induce considerable changes in the salience of echoes from a nearby lateral wall when the sound emission is directed either towards or away from that wall. As a consequence, performance was significantly worse in close proximity of the lateral wall than in the middle of the corridor in Experiment 1.2. Results from conditions 3 and 4 with initial orientations of −10° and +10°, respectively, show that the negative effect of orientation jitter indeed results from orientation uncertainty and not from a deviation from the 0° orientation alone.

Wallmeier and Wiegrebe [23] have shown that free head rotations facilitate sonar-guided directional orientation in humans. This indicates that in the current experiments, head rotations might also have helped subjects to determine their orientation in the virtual corridor and thereby to overcome the orientation uncertainty due to the jitter and to disentangle whether variations in echo-acoustic cues originated from distance changes or from orientation changes.

Concerning the perception of sound source distance, Speigle and Loomis [42] found only little influence of moving compared to stationary listening, whereas Ashmead et al. [43] report a significant effect. In the former study, the intensity of the sound source was held constant, and therefore intensity changes might have provided a sufficiently robust cue for sound source distance, even without dynamic cues. In the latter study, the intensity of the sound source was varied, which might have forced subjects to rely on additional dynamic cues. These findings are similar to the results from the current experiments with and without orientation jitter, respectively. They provide further evidence for the above interpretation, namely that exploratory head rotations do not necessarily enhance echo-acoustic distance discrimination directly, but improve performance under non-optimal conditions, i.e. when static cues are not sufficiently robust.

### The representation of auditory space

Several studies have shown that blind people are able to generate and maintain accurate representations of auditory space without calibration through visual cues [21], [44], [45], [46]. A common model used to explain these findings is the calibration of auditory space by audiomotor feedback [21], [45]. However, this model does not conclusively explain the supra-normal performance of blind subjects in auditory distance estimation in far space [46], which cannot be attributed to the simultaneous perception of self-motion and systematic changes in auditory stimuli. Instead, Kolarik et al. [3] hypothesized that echolocation "may aid in the calibration of auditory space and especially of distance."

The current findings support this hypothesis by demonstrating that blind and blindfolded sighted subjects can use echolocation for distance discrimination with high acuity both in near and in far space. In fact, the observed acuity for echolocating distance is higher than the acuity found in previous studies on the discrimination of sound source distance: For totally blind subjects, Kolarik et al. [47] report sound-source discrimination thresholds of around 0.8 and 1.5 m at the 79.4% correct level for reference distances of 2 and 5 m, respectively. For sighted subjects, thresholds were even larger. In comparison with the current results, this shows that echolocation allows for relatively accurate distance perception, and hence might indeed play an important role in the calibration of auditory space.

## Conclusion

In a formal 2AIFC experiment, we demonstrate that both blind and blindfolded sighted human subjects can use echolocation to discriminate between different distances to the walls of an enclosed space, with reference distances ranging from 0.75 to 4 m. Notably, distance discrimination acuity in the current echolocation experiments was higher than the acuity observed in previous experiments on sound source distance. This indicates that in comparison to other sensory modalities, echolocation provides relatively reliable distance cues and hence may play an important role in depth perception.

Moreover, our experiments shed light on the factors that determine whether room reverberations and additional echoes impede or improve echolocation performance in a specific task: Whereas Wallmeier et al. [5] have shown that additional echoes make it more difficult to assess directional information of a target echo, the current results demonstrate that the availability of distance information is much less affected, or even enhanced, depending primarily on the relative distance of the subject to the target reflector and to the additional reflector. Finally, we have shown that free head rotations during echolocation can improve distance discrimination performance in complex stimulus settings, e.g. when the subject's orientation is varied relative to the target reflector. However, head movements do not necessarily provide a benefit over static echolocation from an optimal single orientation.

In summary, the current study shows that accurate distance perception is an important functional benefit of echolocation in humans, which may also play a major role in the calibration of auditory space representations.

## Supporting Information

S1 File **Recording of the binaural room impulse responses**
(PDF)

S2 File **Analysis of the echolocation sounds.**
(PDF)
