# Supplementary material for: Ranging in Human Sonar: Effects of Additional Early Reflections and Exploratory Head Movements
Source: PLoS One. 2014 Dec 31;9(12):e115363. doi: 10.1371/journal.pone.0115363 (PMC4281102; doi:10.1371/journal.pone.0115363)
Supplement: S1 File — Recording of the binaural room impulse responses (PDF) [file pone.0115363.s001.pdf]

Electronic supplementary material:

Recording of the binaural room impulse responses

For the BRIR recordings, a head-and-torso simulator (HATS, *B&K 4128C, Brüel & Kjaer Instruments, Naerum, Denmark*) was used as the core part of a custom built mobile recording setup. The HATS was attached to a computer controlled turntable which was mounted on a small wooden cupboard with wheels. The recording was controlled via a notebook connected to an external soundcard (*ProFire 610, M-Audio, Willich, Germany*).

The corridor was acoustically excited with a 10 s logarithmic sine sweep with a frequency range from 200 to 20000 Hz. The sweep was created with Matlab (*The MathWorks, Inc., Natick, USA*), amplified (Stereo Amplifier A-109, *Pioneer Electronics, Willich, Germany*), and transmitted to the loudspeaker in the mouth of the HATS. The emitted signal and its reflections were then recorded via the microphones in the ear-canals of the HATS and amplified with a *Brüel & Kjaer Nexus* conditioning amplifier. Playback and recording were implemented with SoundMexPro (*HörTech GmbH, Oldenburg, Germany*). The frequency response of the mouth simulator was digitally equalized. With an overall height of 180 cm, the recording setup was appropriate to simulate a human adult in upright position that actively vocalizes with his mouth and perceives his own vocalizations both directly from the mouth to the ears (direct sound) and from the mouth via reflections to the ears (echo).

To extract the BRIRs, the emission and the binaural recording were cross-correlated and afterwards filtered to compensate for the logarithmic sweep. This procedure was used to acquire a representative database of BRIRs for positions along the midline of the corridor and in close proximity of 65 cm to the right lateral wall, with a longitudinal resolution of 25 cm. At each position, measurements were conducted for 90 orientations with an angular resolution of 4°. Finally, the BRIRs were interpolated (by linear interpolation of the linear magnitude spectra and unwrapped phase spectra) to increase the angular resolution from 4° to 0.2°, and the longitudinal resolution from 25 cm to 1 cm.
