# Supplementary material for: Ranging in Human Sonar: Effects of Additional Early Reflections and Exploratory Head Movements
Source: PLoS One. 2014 Dec 31;9(12):e115363. doi: 10.1371/journal.pone.0115363 (PMC4281102; doi:10.1371/journal.pone.0115363)
Supplement: S2 File — Analysis of the echolocation sounds. (PDF) [file pone.0115363.s002.pdf]

Ranging in human sonar: Effects of additional early reflections and exploratory head movements

Ludwig Wallmeier and Lutz Wiegrebe

Analysis of the echolocation sounds

The subjects' vocalizations during the echolocation experiments were recorded and analysed with custom Matlab programs in terms of the following parameters:

The positions of the calls within a recording were determined by identifying the local maxima in the recording's Hilbert envelope that exceeded a certain threshold (mean amplitude of the whole recording plus three times the standard deviation of the amplitude). Clipped calls and calls starting within the last 50 ms of the interval were excluded from further analysis. The duration of a call was quantified as the length of the time interval that contained 95 % of the energy. As a measure for its sound level, the sound pressure level (SPL) in dB over a temporal window of 85 ms centred on this time interval was calculated. The spectral content was analysed in terms of a call's loudest frequency (peak frequency) and in terms of its high- and low-pass frequencies, defined as the cut-off frequencies at -15 dB below and above the peak frequency. The results of the sound analysis is shown in the figure below.

In the current experiments, subjects were allowed to choose any kind of echolocation call, as long as they produced it with the mouth. After a few training sessions, all subjects ended up emitting short tongue clicks, and continued to do so during the whole data acquisition. The clicks typically had a duration between 2 and 8 ms. The sound pressure levels ranged from about 75 to 95 dB SPL as measured at the headset microphone. The clicks had a relatively high peak frequency of around 2 to 8 kHz. The cut-off frequencies at -15 dB below and above the peak frequency (high-pass and low-pass frequencies) were around 1 to 5 kHz and around 4 to 12 kHz, respectively.

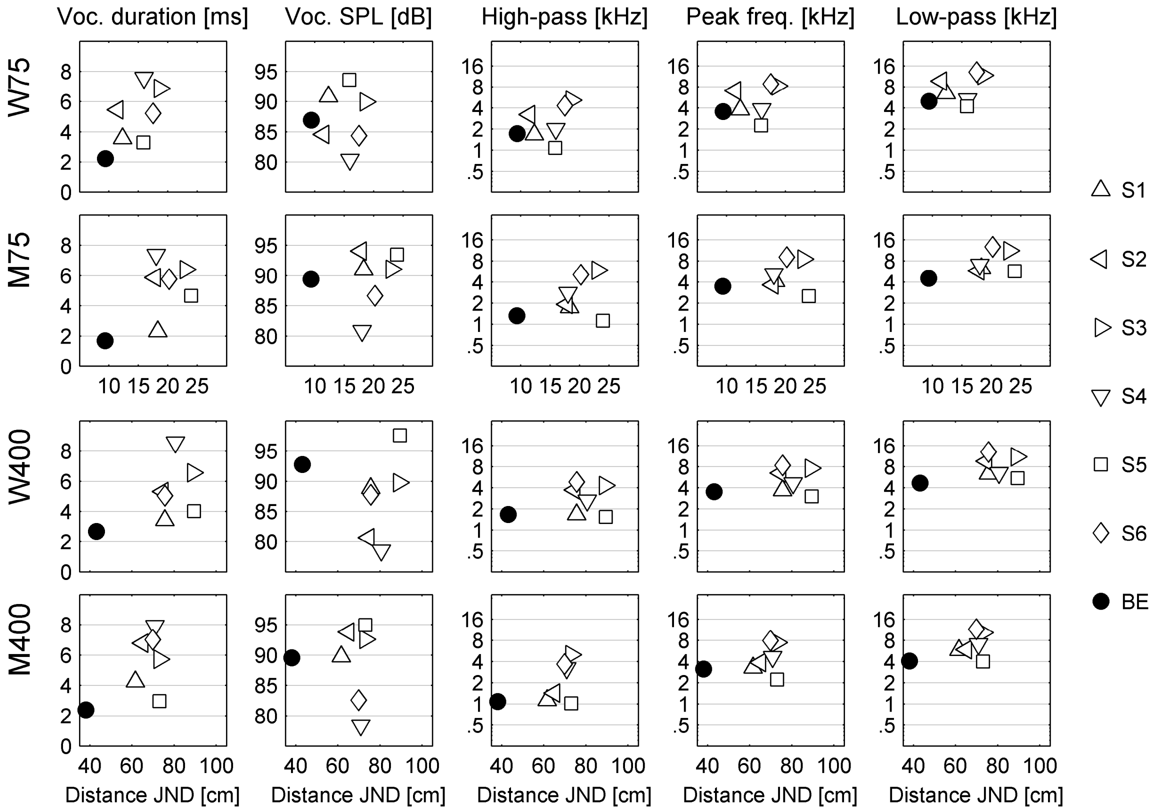

Figure: Acoustic analysis of the calls emitted by the subjects to solve the psychophysical task, in relation to their performance in Experiment 1. The columns represent individual averages of different acoustic parameters. The y-axis labels correspond to the column titles. The acoustic parameters of the individual vocalizations are relatively stable across different reference positions. This indicates that subjects did not adjust their vocalizations to the reference positions.
